# Supplementary material for: The structure of a plant-specific partitivirus capsid reveals a unique coat protein domain architecture with an intrinsically disordered protrusion
Source: Commun Biol. 2021 Oct 6;4:1155. doi: 10.1038/s42003-021-02687-w (PMC8494798; doi:10.1038/s42003-021-02687-w)
Supplement: Supplementary file 1 — Supplementary Information [file 42003_2021_2687_MOESM1_ESM.pdf]

Supplementary Information

The Structure of a Plant-Specific Partitivirus Capsid Reveals a Unique Coat Protein Domain Architecture with an Intrinsically Disordered Protrusion

Matthew Byrne[1], Aseem Kashyap[2], Lygie Esquirol[2], Neil Ranson[1], and Frank Sainsbury[2,3]

[1] Astbury Centre for Structural Molecular Biology, Faculty of Biological Sciences, University of Leeds, Leeds, UK
[2] Centre for Cell Factories and Biopolymers, Griffith Institute for Drug Discovery, Griffith University, Nathan, QLD 4111, Australia
[3] Synthetic Biology Future Science Platform, Commonwealth Scientific and Industrial Research Organization (CSIRO), Brisbane, QLD 4001, Australia

Supplementary Figure 1 – VLPs and SDS-PAGE

Supplementary Figure 2 – Local resolution and FSC

Supplementary Figure 3 – Monomer alignment and b-factors

Supplementary Figure 4 – PCV coat protein organisation

Supplementary Figure 5 – Multiple sequence alignment of Deltapartitivirus coat proteins

Supplementary Figure 6 – Alignment for all identified long-form CPs

Supplementary Figure 7 – Disorder prediction for all identified long-form CPs

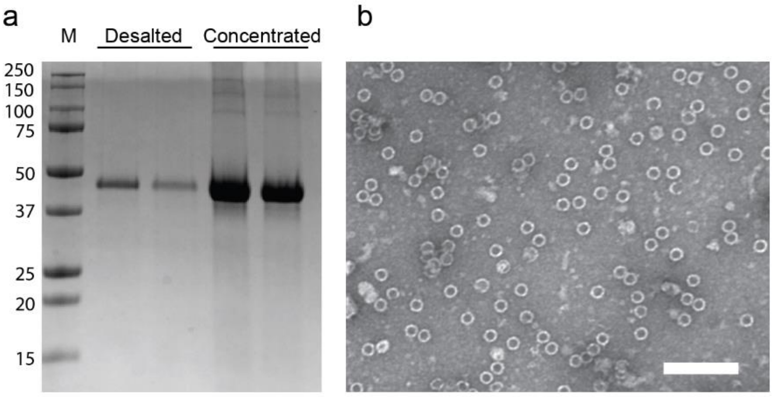

c

MGDRVNAQDDDTVVPHQAPLQPAALQQDLTRSADYLLDNVRIGNHRQRYDKYRRYVLLRS
SEIFTSLVAIYAHIFSSYWQHFRRFTDQFQAPTGVQLPTFVARVYISTWLHDLYCSIREA
TRSISPLAFNERYSYELLPYSTEYDPFLAFLSMSIKPTHIQHTPENTLWIPILCENYDWD
RNEANHNPFGITNFTLNSNLFYGLLAILKERKEFKLSTLTTNTIGRPCWLFDWHDNVQVC
AWFPREANFNSQDVTAAYIIGVACTPKLGPSDDDAWKYYASLNSVPTFTPTEPRLTNRRS
YGAYEVRTRETENNYFLPDSLLNIIEDFTATGTTQRRKIRRPSATSASTGAAIIIRDTPG
TASTATTSTTETEVTFPPVIRTKIRDWYYHSRVILELEDNSRTAALRMFIIA

Supplementary Figure 1. Purification of PCV-1 VLPs. **(a)** SDS-PAGE of two gradient fractions from ultracentrifugation showing a single protein following desalting to remove iodixanol and following concentration using ultrafiltration. **(b)** Negative staining transmission electron microscopy of PCV-1 VLPs, bar = 200 nm. **(c)** Peptide coverage (74%) from mass spectroscopy analysis of bands cut from (A) shows presence of the N-terminal and internal disordered regions. Highlighted sequences (green) indicate peptide coverage and the underlined sequences show regions of disorder.

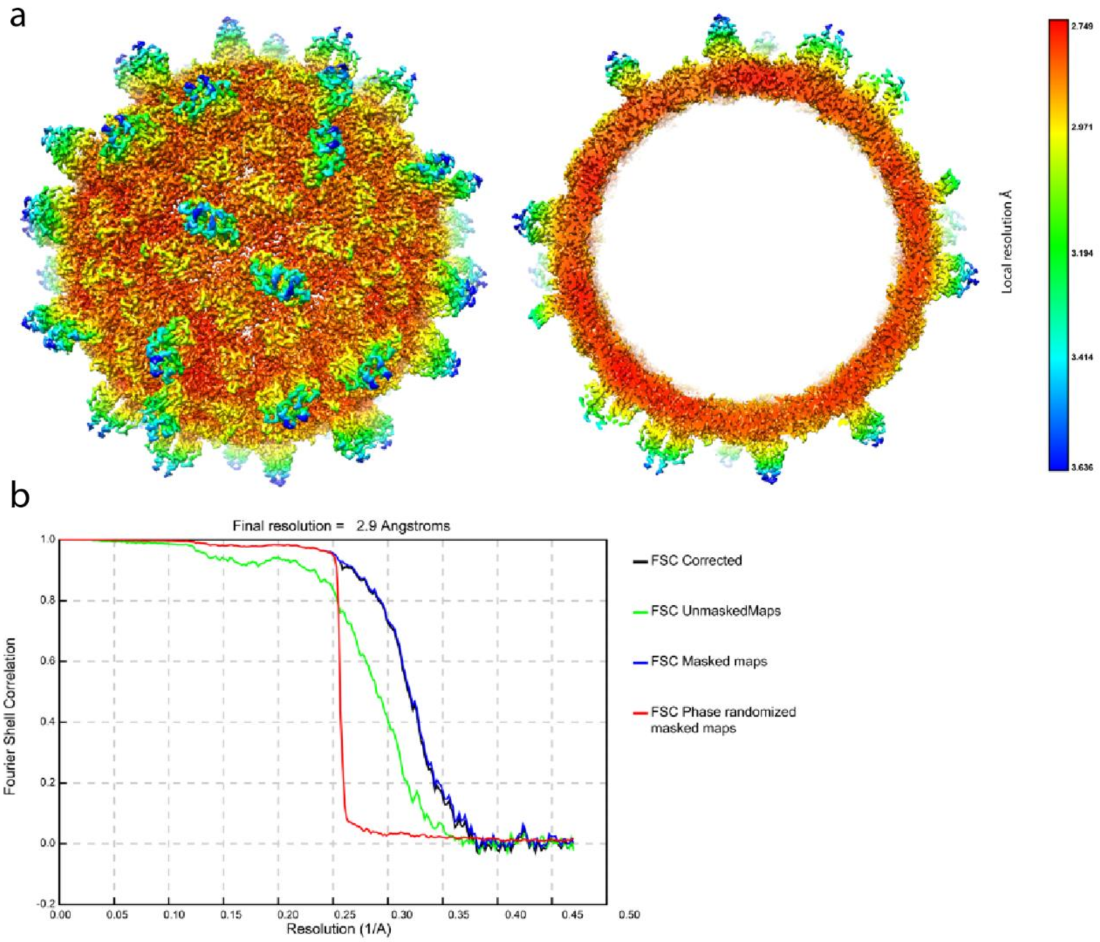

Supplementary Figure 2. PCV-1 VLP reconstruction local resolution and Fourier shell correlation. **(a)** Local resolution filtered map of PCV-1 VLP coloured according to resolution, **(Left)** capsid surface, **(Right)** central slice. **(b)** Fourier shell correlation – the resolution that corresponds to an FSC coefficient of 0.143 is 2.9 Å.

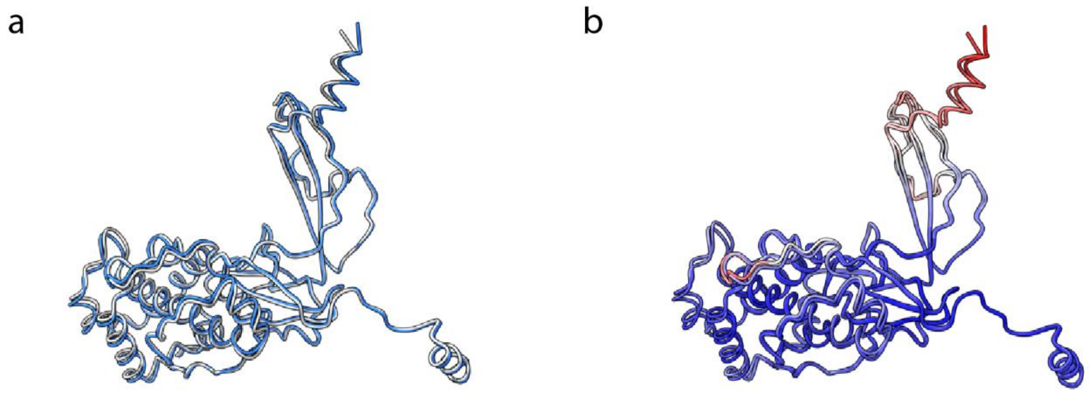

Supplementary Figure 3. Superposition of chain A and chain B from a single asymmetric unit of PCV1. **(a)** Superposed PCV monomers, shown as backbone ribbons for clarity. Coloured according to chain with chain A coloured blue, and chain b coloured grey. **(b)** Superposed PCV monomers, shown as backbone ribbons for clarity. Coloured according to b-factor, from low (blue) to high (red).

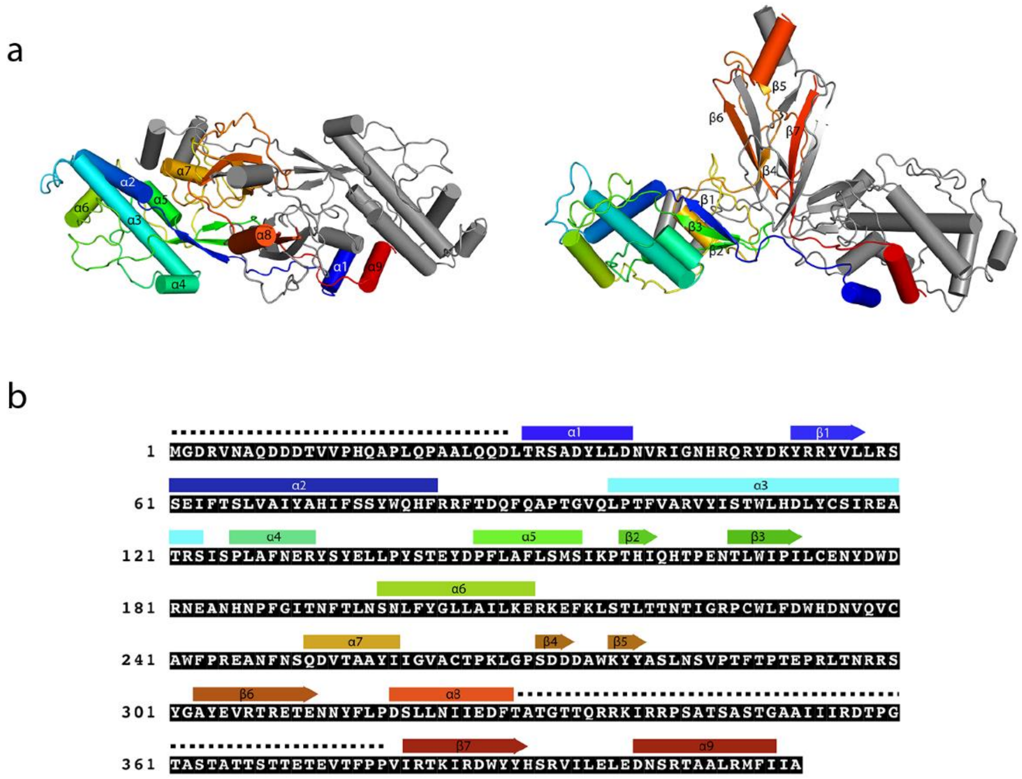

Supplementary Figure 4. PCV coat protein composition. **(a)** PCV CP shown in cartoon form with monomer A coloured from N (blue) to C terminus (red). Monomer B is coloured grey for clarity. **(b)** PCV CP sequence labelled with secondary structure elements. Regions of disorder, that are not present in the final 3D reconstruction are denoted by a dashed line.

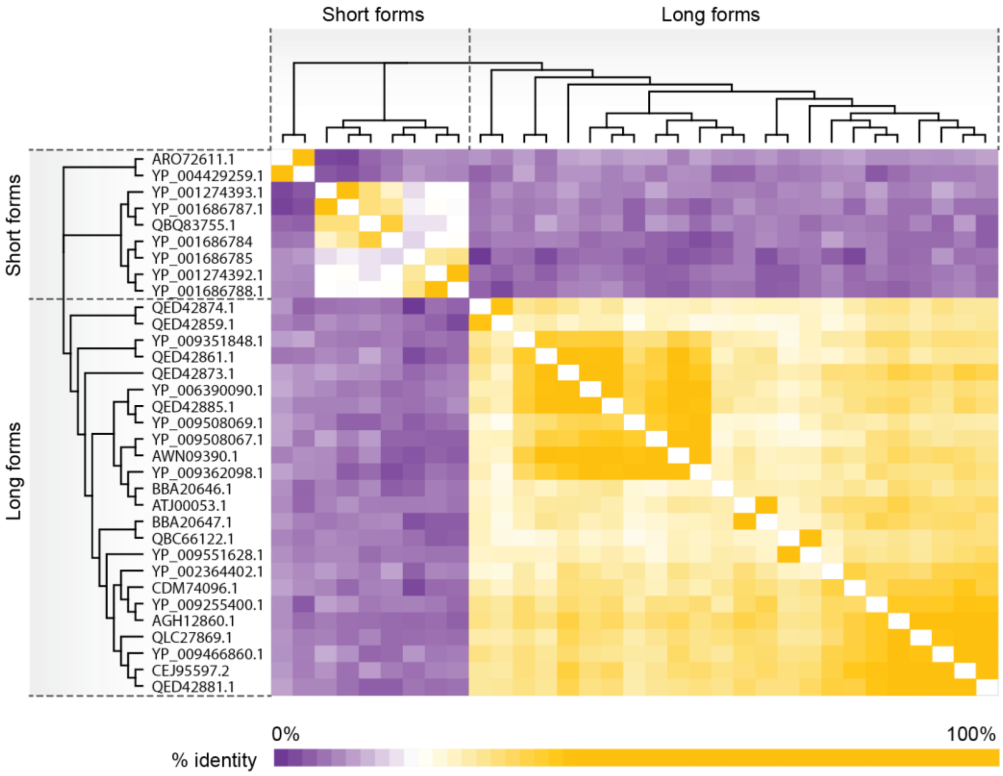

Supplementary Figure 5. Sequence comparison of Deltapartitivirus coat proteins. Colour coded distance matrix based on a multiple sequence alignment performed using Clustal OMEGA[1] and the corresponding cladogram of the neighbour-joining tree. Sequence comparisons shows that there are two distinct forms of the coat protein among the deltapartitiviruses, a long form (374-483 aa) and a short form (335-348 aa). Between the long forms there is 20-91% pairwise identity with no more than 12% similarity with short any form coat proteins. Between the short form viruses there is 6-70% pairwise similarity.

There are five members of the deltapartitivirus (NCBI:txid1511810) recognised by the ICTV[2]. Three of these have the long form of the coat protein; Pepper cryptic virus 1 (PCV-1), Pepper cryptic virus 2 (PCV-2), and Beet cryptic virus 2 (BCV-2) that has two genomic segments encoding putative coat proteins. In addition, the ICTV notes four related viruses that are unclassified, of which one has the long form coat protein; Persimmon cryptic virus. The NCBI also lists three unclassified deltapartitiviruses (NCBI:txid1985162), of which one has the long form coat protein; Medicago sativa deltapartitivirus 1. PSI-BLAST using the PCV-1 coat protein identifies a further 18 viral sequences related to the long form coat proteins, and which are themselves long form coat proteins. The single exception to the length demarcation is a Citrullus lanatus partitivirus coat protein at 337 aa[3].

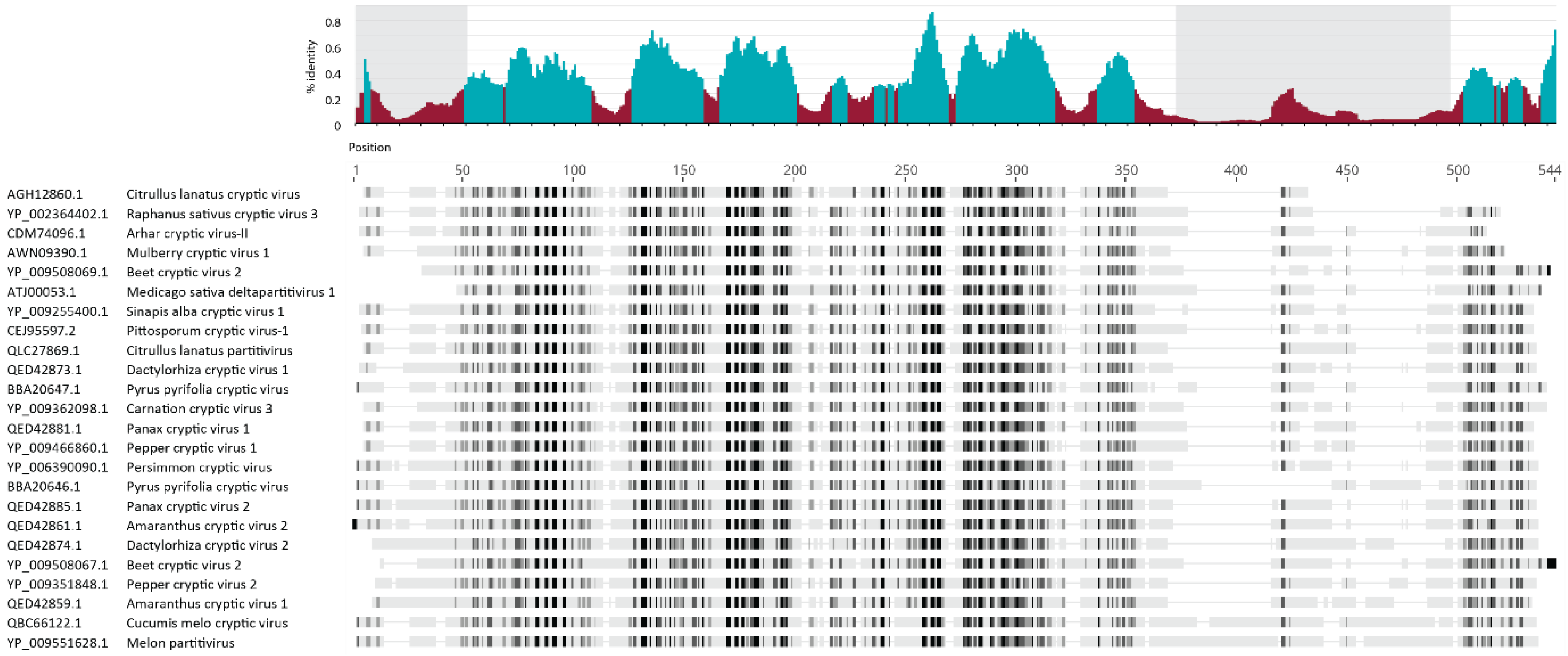

Supplementary Figure 6. Alignment of CP sequences of all 24 identified long form deltapartitivirus CP sequences. Shaded regions correspond to the unresolved regions of PCV-1 CP and positions coloured cyan correspond to ≥ 25 % identity using a 10-position rolling average. The alignment is ordered according to the length of the CP sequence.

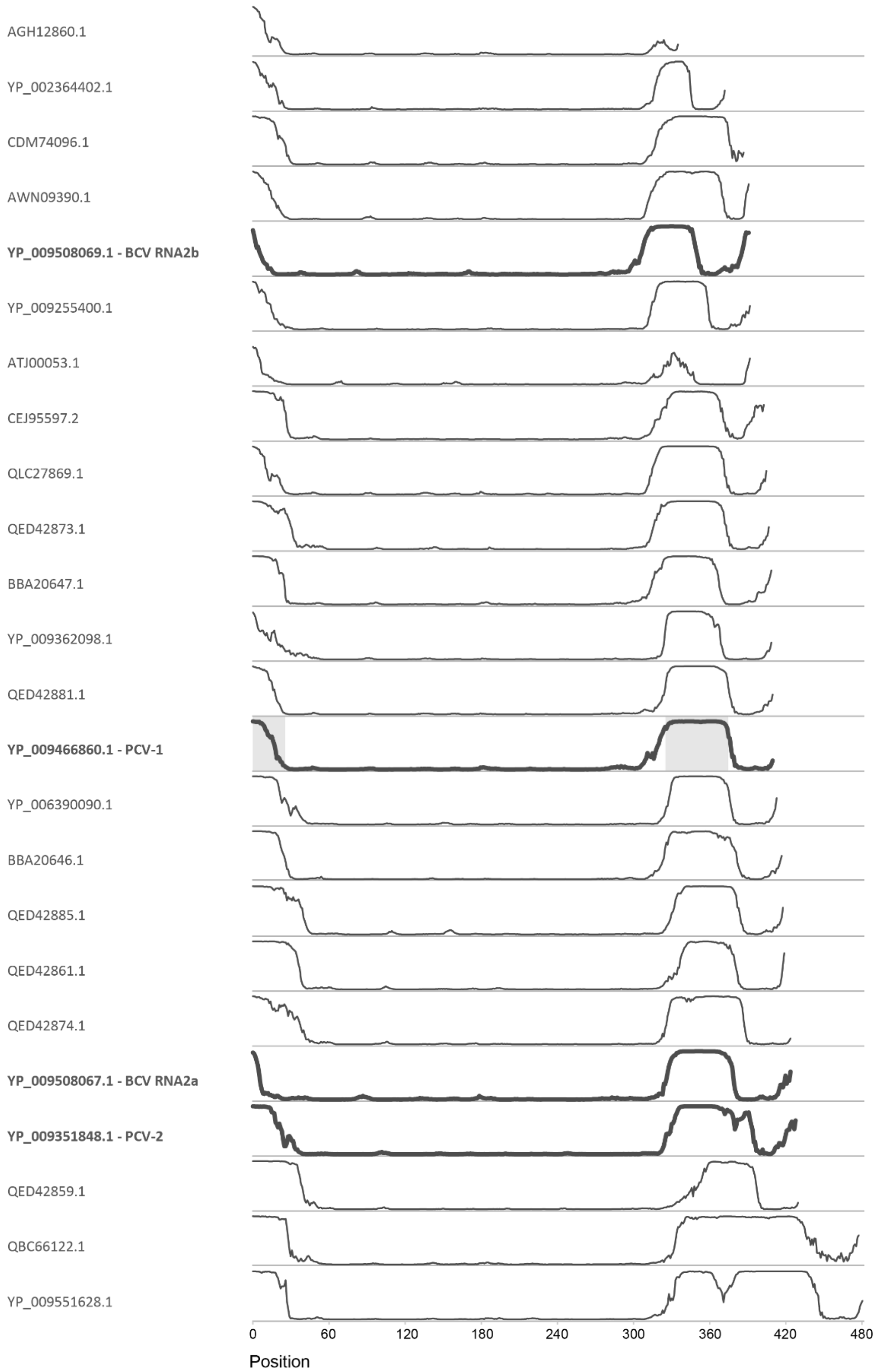

Supplementary Figure 7. Predicted disorder regions for the long form deltapartitiviruses. Predictions using NetSurfP-2.0[4] show that the putative region of disorder on a scale 0 to 1 starts in approximately the same position for all 24 identified long form deltapartitivirus CP sequences, which are presented in order of the sequence length. Bold series represent those among the ICTV-recognised *deltapartitivirus* genus[2] and the experimentally determined disorder for the PCV-1 CP is shaded.

Supplementary References:

1 Sievers, F. & Higgins, D. G. Clustal Omega for making accurate alignments of many protein sequences. *Protein Science* **27**, 135-145, doi:https://doi.org/10.1002/pro.3290 (2018).

2 Vainio, E. J. *et al.* ICTV Virus Taxonomy Profile: Partitiviridae. *J Gen Virol* **99**, 17-18, doi:10.1099/jgv.0.000985 (2018).

3 Sela, N., Lachman, O., Reingold, V. & Dombrovsky, A. A new cryptic virus belonging to the family Partitiviridae was found in watermelon co-infected with Melon necrotic spot virus. *Virus Genes* **47**, 382-384, doi:10.1007/s11262-013-0937-8 (2013).

4 Klausen, M. S. *et al.* NetSurfP-2.0: Improved prediction of protein structural features by integrated deep learning. *Proteins: Structure, Function, and Bioinformatics* **87**, 520-527, doi:https://doi.org/10.1002/prot.25674 (2019).
